# Uncomplicated Spontaneous Rupture of the Pancreatic Pseudocyst into the Gut – CT Documentation: A Series of Two Cases

**DOI:** 10.4103/1319-3767.48975

**Published:** 2009-04

**Authors:** Mohammed F. Mir, Feroze Shaheen, Tariq A. Gojwari, Manjeet Singh, Pervez Nazir, Shafeeq Ahmad

**Affiliations:** Department of Radiodiagnosis, Sher-i-Kashmir Institute of Medical Sciences, Srinagar, Kashmir, India

**Keywords:** Pancreatic pseudocyst, spontaneous rupture, computed tomography

## Abstract

Spontaneous rupture of the pancreatic pseudocyst into the surrounding hollow viscera is rare and, may be associated with life-threatening bleeding. Such cases require emergency surgical intervention. Uncomplicated rupture of pseudocyst is an even rarer occurrence. We present herein two cases of uncomplicated spontaneous rupture of a pancreatic pseudocyst into the stomach with complete resolution.

Spontaneous rupture of the pancreatic pseudocyst into the surrounding hollow viscera is rare and, whenever it occurs, is associated with life-threatening bleeding.

We present a series of two cases of uncomplicated spontaneous rupture of a pancreatic pseudocyst into the stomach with complete resolution.

## CASE REPORT

### Case 1

**Figure 1:**
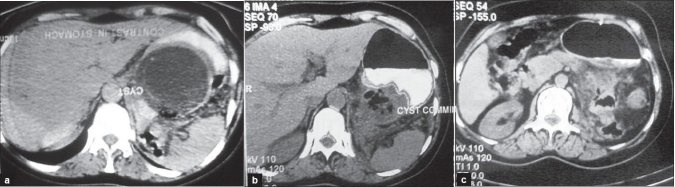
(a) Computed tomography of the abdomen showing the retrogastric cyst compressing the contrast-filled stomach. (b) CT of the abdomen showing the retrogastric cyst emptying into the contrast-filled stomach. (c)CT of the abdomen showing complete evacuation of the cyst

A 44-year-old nonalcoholic male presented with an epigastric mass and postprandial fullness. The patient had been treated as acute pancreatitis by conservative management 2 months earlier. Contrast enhanced computed tomography (CECT) revealed a large retrogastric cystic mass with normal enhancement of the surrounding pancreatic tissue measuring 7.6 × 7.5 cm [Figure 1a]. Seven days after admission, the patient complained of vomiting, diarrhea, and fever. Repeat computed tomography (CT) revealed the retrogastric cyst communicating with the contrast-filled stomach through the fistula in the posterior gastric wall [Figure 1b]. During the hospital stay for the next 3 days, the patient did not develop any complication. Repeat CECT of the abdomen on discharge revealed complete resolution of the cyst [Figure 1c].

### Case 2

**Figure 2:**
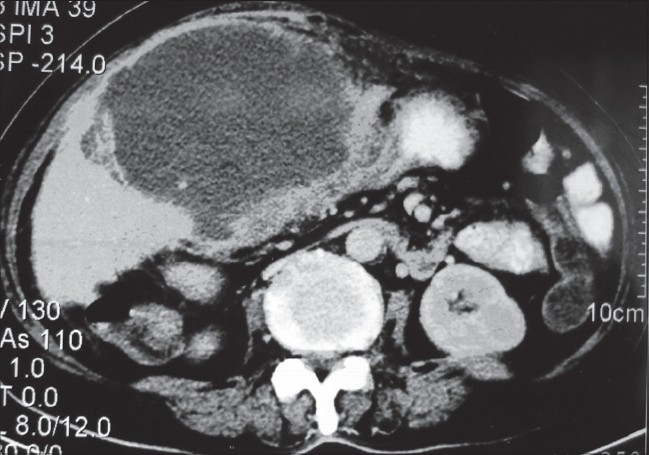
Large pseudocyst at the porta extending into the omental bursa and root of the mesentery

A 70-year-old female with a previous history of pancreatitis presented to the emergency service as a palpapable abdominal mass. Ultrasonography followed by CECT of the abdomen revealed a large pseudocyst measuring 12 × 8 cm at the porta extending into the omental bursa [Figure 2]. The patient was planned for catheter drainage of the cyst. Three days later, the patient complained of sudden abdominal pain and vomiting. After immediate management, the patient was sent for a CECT examination.

**Figure 3:**
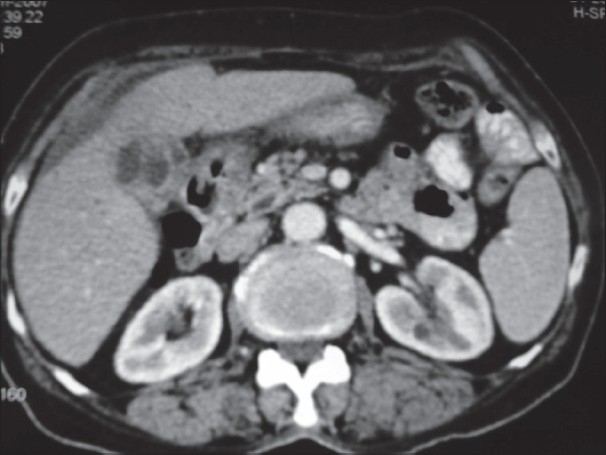
Computed tomography scan after rupture, which shows near-complete resolution of the large pseudocyst into the omental bursa with decompression of the biliary tree with a very small residual collection

A CT scan showed near-complete resolution of the large pseudocyst in the omental bursa with decompression of the biliary tree [Figure 3]. Residual collection measured 2 × 2 cm. For the next 72 h, the patient's condition remained stable and she was discharged. Follow-up CT after 2 weeks showed complete resolution of cyst.

## DISCUSSION

Many cases of spontaneous resolution of pancreatic pseudocyst have been reported in the literature. However, all the patients had evidence of acute gastrointestinal bleed, extension into the biliary tree, and pelvicalceal system in one case. Both our patients reporting the above did not develop any such complication.

In a longitudinal study over 30 months, Mehta ***et al.***[1] found that pseudocysts less than 7.5 cm in diameter with a volume of less than 250 ml and with absence of internal debris were associated with spontaneous resolution over an average duration of 5 months whereas cysts larger than 7.5 cm in size or >250 ml in volume needed surgical endoscopic intervention.

Spontaneous rupture of the pancreatic pseudocysts is known to occur into the stomach, duodenum, biliary tract, renal collecting system, colon, and bronchial tree.[2] However, most of these spontaneous ruptures are associated with bleeding complications needing emergency surgical intervention.[3] There are very few reports of uncomplicated rupture of pseudocysts in the literature,[4] like the ones reported here.

## References

[1] Mehta R, Suva Rana D (2004). Natural course of asymptomatic pancreatic pseudocyst: A prospective study. Indian J Gastroenterol.

[2] Tanaka A, Takeda R, Utsunomiya H (2000). Severe complications of mediastinal pancreatic pseudocyst: Report of oesophago-bronchial fistula and haemothorax. J Hepatobiliary Pancreat Surg.

[3] Urakami A, Taunodo, Kubozoe T, Takeo T, Yamashita K, Imai H (2002). Rupture of a bleeding pancreatic pseudocyst into the stomach. J Hepatobiliary Pancreat Surg.

[4] Willard MR, Schafer HA (1982). Resolution of pancreatic pseudo-cyst by spontaneous rupture into stomach. South Med J.

